# Supplementary material for: The weathering process of carbonatite: weathering time
Source: PeerJ. 2023 Jul 31;11:e15793. doi: 10.7717/peerj.15793 (PMC10399557; doi:10.7717/peerj.15793)
Supplement: Supplemental Information 2 [file peerj-11-15793-s002.docx]

**Table S1**

Types and species of epiphytes in limestone with different weathering times.

| Weathering Time | Types of epiphytes | Species |
| --- | --- | --- |
| 22 | Only lichens exist | *Caloplaca holocarpa* (Athallia holocarpa) |
| 23 | Only lichens exist | *Caloplaca holocarpa* (Athallia holocarpa), *Caloplaca flavovirescens* |
| 37 | Lichens and mosses coexist | *Aspicilia cinerea*, *Eurohypnum leptothallum* (Müll. Hal.) Ando, H*ypnum plumaeforme* Wils. |
| 39 | Lichens and mosses coexist | *Caloplaca flavovirescens, Hypnum plumaeforme* Wils. |
| 58 | Lichens and mosses coexist | *Aspicilia cinerea*, *Caloplaca flavovirescens, Hypnum plumaeforme* Wils. |
| 72 | Only mosses exist | *Brachythecium fasciculirameum* Müll. Hal., *Meteorium buchananii* (Brid.) Broth. in Engl. & Prantl |
| 88 | Only mosses exist | *Thuidium cymbifolium, Climacium dendroides* Web.et Mohr |
| 104 | Only mosses exist | *Tortella humilis* (Hedw.) Jenn., *Weisia controvers* Hedw., *Thuidium cymbifolium,* |
| 133 | Only mosses exist | *Polytrichum commune* Hedw. |
| 161 | Only mosses exist | *Bryum argenteum* Hedw., *Heddigia ciliate*, *Bryum caespiticium* |
