# Supplementary material for: The weathering process of carbonatite: weathering time
Source: PeerJ. 2023 Jul 31;11:e15793. doi: 10.7717/peerj.15793 (PMC10399557; doi:10.7717/peerj.15793)
Supplement: Supplemental Information 3 [file peerj-11-15793-s003.docx]

**Table S2**

Statistical table of the relationship between each index and weathering time.

| Items | Slope | *SE* | *R^2^* | Adj *R^2^* | *p* | Model |
| --- | --- | --- | --- | --- | --- | --- |
| *CaO* | -36.93 | 3.94 | 0.71 | 0.67 | 0.002** | *y=a+bx* |
| *MgO* | 115.70 | 25.84 | 0.018 | -0.105 | 0.7097 | *y=a+bx* |
| *Al_2_O_3_* | 495.25 | 103.83 | 0.21 | 0.11 | 0.1844 | *y=a+bx* |
| *Fe_2_O_3_* | 454.63 | 103.86 | 0.01 | -0.11 | 0.7793 | *y=a+bx* |
| *SiO_2_* | 400.96 | 73.26 | 0.29 | 0.21 | 0.1031 | *y=a+bx* |
| *TiO_2_* | 672.72 | 152.91 | 0.21 | 0.09 | 0.2181 | *y=a+bx* |
| *AC* | 0.088 | 0.0097 | 0.75 | 0.72 | 0.0012** | *y=a+b×ln(x)* |
| *CIA* | 8.04 | 0.82 | 0.77 | 0.74 | <0.001*** | *y=a+b×ln(x)* |
| *Lc* | 0.32 | 0.036 | 0.72 | 0.68 | 0.0019** | *y=a+b×ln(x)* |
| *I_mob_* | 0.77 | 0.12 | 0.73 | 0.70 | 0.0016** | *y=a+b×ln(x)* |
| *W_s_* | 34.55 | 3.50 | 0.09 | -0.03 | 0.41 | *y=a+bx* |
